# Editorial: Modulation of human immune parameters by anticancer therapies, volume II

**DOI:** 10.3389/fimmu.2023.1249099

**Published:** 2023-07-26

**Authors:** Il-Kang Na, Ulrich Sack, Silvia Sánchez-Ramón

**Affiliations:** ^1^Berlin-Brandenburg Center for Regenerative Therapies, Charité Medical University of Berlin, Berlin, Germany; ^2^Medical Clinic with a Focus on Hematology, Oncology and Tumor Immunology, Charité University Medicine Berlin, Berlin, Germany; ^3^German Cancer Consortium (DKTK), Charite - Universitätsmedizin Berlin, corporate member of Freie Universität Berlin and Humboldt-Universität zu Berlin, Berlin, Germany; ^4^Experimental and Clinical Research Center, Charite University Medicine Berlin, Berlin, Germany; ^5^Medical Faculty, Institute of Clinical Immunology, Leipzig University, Leipzig, Germany; ^6^Department of Clinical Immunology , San Carlos University Clinical Hospital, Madrid, Spain

**Keywords:** immunotherapy, immuno-oncology, secondary immune defects, biomarker, tumor microenvironment

In contrast to the first volume of this special Research Topic (https://www.frontiersin.org/research-topics/10713/modulation-of-human-immune-parameters-by-anticancer-therapies#articles), this time the focus is not on methodological aspects, but rather on therapy-induced alterations on the tumor microenvironment and immune system of cancer patients.

We refer here to other Research Topics in Frontiers that address the use of genetically modified cells and also consider the immunological consequences of monoclonal antibodies, which are now widely used. Recent therapeutic methods based on immunological principles are rapidly changing the possibilities for treating and in some cases even curing patients with oncological diseases. In addition to the desired anti-tumor effect, such therapeutic approaches always have an impact on the normal function of the immune system. It is therefore no coincidence that Frontiers in Immunology is publishing for the second time a thematic issue on the challenges of the modulation of human immune parameters by anti-cancer therapies and conversely, of taking advantage of immune cells to eliminate cancer cells. This Research Topic comprises eight articles addressing a wide variety of aspects in immuno-oncological therapies.

Several authors address the tumor microenvironment and the connective tissue and infiltrating immune cells localized around the tumor as potential new avenues in immunotherapy.

Eskandari-Malayeri and Rezaei summarize what is known about immune checkpoint inhibitors as mediators of immune suppression by cancer-associated fibroblasts of the tumor environment. There is growing evidence that targeting tumor cells while neglecting the surrounding environment is not sufficient to defeat cancer. Fibroblasts are important sentinels of stroma that are activated under certain conditions in the TME, such as oxidative stress and local hypoxia, and play a prominent role in physically supporting tumor cells and enhancing tumorigenesis. Focusing on the direct and indirect roles of CAFs in the induction of iICPs in the TME and their use in immunotherapy and immunodiagnostics, the authors present in their review the evolving understanding of the immunosuppressive mechanism of CAFs in the TME.

Rupp et al.’s team investigated the prognostic significance of T-cell composition and spatial organization after treatment in soft tissue sarcoma patients treated with neoadjuvant hyperthermic radio(chemo)therapy. Increasing evidence suggests that the tumor immunologic microenvironment plays a central role in determining clinical outcome and response to therapy. Here, the authors investigated the effects of curative multimodal therapy on the T-cell landscape of STS using multiplex immunohistochemistry. The results suggest that the T-cell landscape of STS is altered by multimodal therapy and may influence the clinical outcome of patients.

Umeyama et al. investigated three different mechanisms underlying human γδ-T cell-mediated cytotoxicity against malignant pleural mesothelioma. Hunter reason is the poor response of malignant pleural mesothelioma (MPM) to immune checkpoint inhibitors in some patients. γδ-T cells derived from peripheral blood mononuclear cells may be a therapeutic option based on cytotoxic activity *via* NK receptors, TCRs, and CD16.

Similarly, Wang et al.’s research group discusses tumor-associated macrophages (TAMs) as targets for glioblastoma immunotherapy in context of current research and latest clinical trials. TAMs can directly eliminate tumor cells and enhance the phagocytic ability of immune cells. An abundance of TAMs at the glioblastoma tumor site suggests that immunotherapy targeting TAMs may be a potential treatment modality for this aggressive cancer.

Therapeutic aspects in this volume include three punctual aspects: Immune recovery after HSCT, immune signature after checkpoint inhibition, and neuro/psychoimmunologic aspects.

Heck et al.’s team focussed on immune reconstitution after autologous hematopoietic stem cell transplantation. High-dose chemotherapy followed by autologous hematopoietic stem cell transplantation (auto-HSCT) is a standard treatment for patients with multiple myeloma (MM). Common and potentially fatal side effects after auto-HSCT are infections due to a severely compromised immune system with impaired humoral and cellular immunity. For this purpose, they examined peripheral blood from patients by flow cytometry and with T-cell-dependent (TD) and T-cell-independent (TI) stimulation assays. Functionally, these patients showed impaired TD as well as TI B-cell immune responses. Individual functional responses correlated with quantitative changes in CD19+, CD4+, memory B cells, and marginal zone-like B cells. In addition, extrinsic factors were identified that should be tested as potential biomarkers and may also indicate therapeutic potential.

Berszin et al. investigated cytokine profiles of squamous cell carcinomas of the head and neck under dual immunotherapy with cetuximab and pembrolizumab, two antibodies used for squamous cell carcinomas of the head and neck either as single agents or in combination with cisplatin and other chemotherapeutic agents. This showed great heterogeneity in response to cetuximab, pembrolizumab, and both in combination with and without IFN-γ stimulation. They identified interferon gamma-induced protein 10 as a novel biomarker.

Tumor characterization itself is the subject of only one article here, the characterization of sialylation-related long non-coding RNAs in the paper by Zhou et al.. The aim was to develop a novel signature for predicting prognosis, immune landscape, and chemotherapy response in colorectal cancer. The prognostic significance and biological properties of sialylation-related long noncoding RNAs in colorectal cancer are still unclear. Analysis of the data revealed that sialylation-related lncRNA signature was an independent prognostic factor for overall survival, progression-free survival, and disease-specific survival prediction. The comprehensive analysis indicated that low-risk patients had higher activity of immune response pathways, greater infiltration of immune cells, and higher expression of immune stimulators.

Stress and cancer are the subject of Liu et al. They highlight and review the mechanisms of immune system dysregulation and its management. Persistent chronic stress accelerates tumor development and progression, which has unfavorable effects on clinical outcomes of cancer patients. The best known is the dysfunction of the hypothalamic-pituitary-adrenal (HPA) axis and the sympathetic nervous system (SNS). Through abnormal activation of the neuroendocrine system, stress-related hormones contribute to increased expression of oncogenes, exacerbated chronic inflammation, and impaired immune function.

A better understanding of the changes that occur with therapy will not only allow the identification of secondary defects and dysregulations of the immune system and interactions of immune and tumor cells but could also lead to the discovery of biomarkers or risk stratification markers for adverse clinical outcome and, in the best case, also hint to new therapy targets.

## Author contributions

US, SS-R, and I-KN identified the authors for the Volume II of this topic, supported the publication process, and wrote the editorial. All authors contributed to the article and approved the submitted version.

